# TGFβ3 (*TGFB3*) polymorphism is associated with male infertility

**DOI:** 10.1038/srep17151

**Published:** 2015-11-27

**Authors:** Marek Droździk, Maciej Kaczmarek, Damian Malinowski, Urszula Broś, Anna Kazienko, Rafał Kurzawa, Mateusz Kurzawski

**Affiliations:** 1 Department of Experimental and Clinical Pharmacology, Pomeranian Medical University, Szczecin, 70-111, Poland; 2 Department of Reproductive Medicine and Gynecology, Pomeranian Medical University, Police, 72-010, Poland; 3 VitroLive Fertility Clinic, Szczecin, 71-074, Poland

## Abstract

Factors affecting the blood-testis barrier function may be involved in testicular damage and male infertility. Two cytokines play an important role in the barrier regulation, namely transforming growth factor beta 3 (TGF-β3) and tumor necrosis factor (TNF-α). The aim of this study was to investigate the potential association between TGF-β3 (*TGFB3*) and TNF-α (*TNF*) gene polymorphisms and male infertility. A total of 846 subjects, 423 diagnosed with male infertility and 423 fertile men were enrolled. *TGFB3* (rs2268626:T > C, rs3917158:C > T, rs2284792:A > G, rs2268625:T > C, rs3917187:C > T) and *TNF* (rs1800629:-308G > A) gene polymorphisms were genotyped. No association between *TNF* genotype and infertility was observed. As for *TGFB3*, the genotypes distribution was similar in infertile and fertile men. However, rs2284792 minor allele frequency was significantly higher among infertile subjects. Heterozygous rs2284792 AG genotype was associated with increased odds for infertility [OR = 1.40 (95% CI 1.05–1.86), p = 0.021] and similar results were observed for G allele carrier status [OR = 1.40 (95% CI 1.06–1.84), p = 0.017]. Heterozygosity in *TGFB3* rs3917158 was also associated with the infertility [OR = 1.37 (95% CI 1.01–1.87), p = 0.041]. The *TGFB3* variant genotypes were associated with lower spermatozoa motility parameters in fertile men. The results indicate that variants in *TGFB3* gene may be associated with male infertility. However, the findings require further replication and validation.

Infertility is a common problem affecting one in six couples, and in 50% it is attributed to the male factor resulting from acquired and/or congenital abnormalities, in which 60–75% is found to be idiopathic^1^,^2^. The pattern of familial aggregation of male infertility implies also an important role of genetic factors, that influence a variety of physiological processes including hormonal homeostasis, spermatogenesis, and sperm quality^3^.

Most of male infertility genetic association studies explored factors involved in spermatogenesis. However, it seems reasonably to explore also an impact of genetic determinants of the blood-testis barrier integrity. Factors affecting the barrier function may be directly involved in the development of testicular damage and problems with fertility. The blood-testis barrier is a very restrictive blood-tissue barrier, and creates a specialized microenvironment for development and maturation of germ cells. It consists of three components: anatomical (tight junctions), physiological (transporters) and immunological. The barrier components restrict passage of molecules and cells (anatomical) and regulate movement of substances in or out of the lumen (physiological) as well as limit access by the immune system cells and sequester the majority of autoantigenic germ cells (immunological). The blood-testis barrier is not a static structure, as it undergoes extensive restructuring during spermatogenesis to meet the needs of germ cells and Sertoli cells. The anatomical barrier tight junctions undergo remodeling (opening, closing) to allow adaptation to Sertoli cell changes (but remain static for their function as integrity of the junctions remains intact) and transporter expression also adapts to various demands during spermatogenesis. Disruption of the barrier function and integrity (e.g. by environmental toxicants such as bisphenol A or cadmium) lead to testicular injury and infertility^4^,^5^.

Two crucial cytokines are involved in the regulation of the blood-testis barrier, namely transforming growth factor beta 3 (TGF-β3) and tumor necrosis factor (TNF-α). These cytokines determine the homeostasis of tight junctions and basal ectoplasmic specialization-structural proteins, proteases, protease inhibitors, and other extracellular proteins (e.g. collagen) in the seminiferous epithelium. Some of those molecules are known regulators of focal contacts between the extracellular matrix and other actively migrating cells, such as macrophages, fibroblasts or malignant cells. Experimental studies revealed that administration of IL-1β or TNF-α impair integrity of the blood-testis barrier^6^.

In the testis, TNF-α, a proinflammatory cytokine, is secreted by germ cells, predominantly pachytene spermatocytes and round spermatides as well as Sertoli cells, whereas its receptors are restricted to Sertoli cells^7^. During the assembly of Sertoli cell tight junction-barrier *in vitro*, the amount of TNF-α produced by Sertoli cells declines significantly, indicating that it can downregulate the Sertoli cell tight junction–barrier function. Co-incubation of Sertoli cells with recombinant TNF-α results in disruption of the integrity of tight junction barrier, as shown in experiments quantifying the transepithelial electrical resistance across the Sertoli cell monolayer^7^. The effect could be produced by direct effect of TNF-α on the tight junction-associated structural proteins’ complexes, as significant reduction of occluding level was observed after TNF-α exposure. Besides, it was shown that TNF-α was able to inhibit claudin-11 expression in cultured mouse Sertoli cells. TNF-α may also affect the blood-testis barrier function via activation of metalloproteinase 2 (MMP-2). MMP-2 is a defined factor regulating tight junction structure and function in the Sertoli cell-tight junction barrier^8^. Another possible route through which TNF-α affects tight junction-barrier function is its impact on follicle-stimulating hormone (FSH) level, since the hormone regulates assembly of Sertoli cell tight junction barrier^9^. Furthermore, subsequent studies have shown that TNF-α can mediate its effects via mitogen-activated protein kinase (MAPK) pathways and the integrin-linked kinase (ILK)/glycogen synthase kinase 3 β (GSK-3β)^7^. This in turn affects the homeostasis of proteases and protease inhibitors and subsequently the nature of the extracellular matrix adjacent to the blood-testis barrier^6^. Having in mind the aforementioned findings it is reasonable to undertake studies on associations of the genetic polymorphism of *TNF* gene (coding for TNF-α) with male infertility. It was documented that TNF-α activity depends on the polymorphic alleles of *TNF*. A single nucleotide polymorphism (SNP) in *TNF* gene promoter is a result of guanine for adenine transition (−308G > A). *In vitro* transfection studies revealed higher expression of −308A minor allele in comparison to −308G^10^, that may be responsible for variability of TNF-α concentration in patients with infectious diseases^11^.

Another cytokine which plays an important role in the regulation of the blood-testis barrier function is transforming growth factor-β3 (TGF-β3), which is the most abundant form of TGF-β in the testis. It is produced by Sertoli cells, and its level declines when Sertoli cell tight junction barrier is being assembled *in vitro*^12^. It is also a product of premeiotic germ cells, such as spermatogonia and early spermatocytes. *In vitro* studies documented that recombinant TGF-β3 led to disruption of Sertoli cell monolayer^12^. The disruption of Sertoli cell tight junctions is accompanied by a decline in the protein levels of several tight junction associated proteins, including occluding, ZO-1 and claudin-11 in Sertoli cells. Subsequent studies have shown that TGF-β3 mediates its effects on Sertoli cell tight junctions via the p38 MAPK pathway. More importantly, those observations have been validated *in vivo* using the CdCl_2_-treated rat model of infertility, i.e. an increase of TGF-β3 was paralleled by decreased content of occludin and ZO-1^13^. Recent observations have also documented that TGF-β3 significantly downregulated junctional adhesion molecule-B expression via post-transcriptional and post-translational modulation, and resulted in the disruption of the blood-testis barrier and apical ectoplasmic specializations^14^. Current data indicate an implication of TGF-β3 polymorphism (*TGFB3*) in the pathology of connective tissue. A significant association was found between TGF-β3 gene (*TGFB3*) polymorphism and fibrosis in patients with sarcoidosis (SNP 4875A > G and 17369T > C)^15^ as well as heart fibrosis^16^. Based on the proved biological role of TGF-β3 a study on its polymorphism in male infertility seem to be well established.

## Results

Standard sperm parameters of the study subjects are presented in Table 1. Comparison of sperm characteristics of infertile and fertile men revealed significant differences in all studied parameters. i.e. concentration, total number of spermatozoa, morphology and motility.

**Figure 1:**
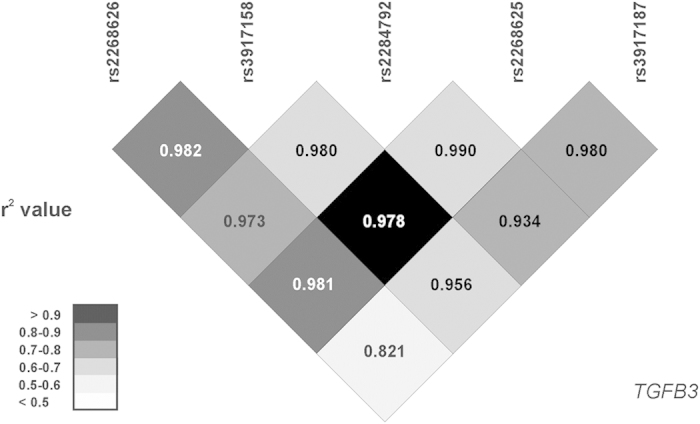
Pairwise LD between the studied *TGFB3* SNPs. Analysis based on genotyping data from all study subjects (n = 846). Numbers represent D’, r^2^ values (square correlation coefficient) are color-coded.

Distribution of all the genotypes was in concordance with Hardy-Weinberg equilibrium, both in fertile and infertile group (p > 0.1). No association was observed between *TNF* genotype and male infertility (Table 2). As for *TGFB3*, the studied genotypes’ distribution was similar in infertile and fertile men. However, minor allele frequency (MAF) was higher among infertile subjects in case of all the studied SNPs, reaching statistical significance level for *TGFB3* rs2284792 (p = 0.025). Heterozygous rs2284792 AG genotype was associated with increased odds for infertility [OR = 1.40 (95% CI 1.05–1.86), p = 0.021] and similar results were observed for G allele carrier status [AG or GG genotype: OR = 1.40 (95% CI 1.06–1.84), p = 0.017]. As for *TGFB3* rs3917158, only in the case of heterozygous subjects, an association with infertility was significant [OR = 1.37 (95% CI 1.01–1.87), p = 0.041]. For all other SNPs, only marginally significant associations were observed when dominant model was applied (p < 0.1, Table 2). As all the analyzed polymorphisms within the *TGFB3* gene are non-coding SNPs in strong linkage disequilibrium (Fig. 1), they should be only considered as markers, not directly influencing gene expression nor protein function, with rs2284792:A > G SNP as the one potentially the most predictive.

Due to strong linkage between the *TGFB3* SNPs, haplotype frequencies were assessed, in both infertile and control groups. It was revealed, that 5 SNPs form two most frequent haplotypes T_C_A_T_C (estimated frequency of 0.750 among infertile and 0.786 among fertile man), and C_T_G_C_T (0.165 and 0.139, respectively, Table 3), and only 3 more variants were present in the study population with frequency > 1% (from 32 potentially possible combinations for 5 *loci*). However, frequencies of the haplotypes did not differ significantly between infertile cases and controls (Table 3).

When the studied *TGFB3/TNF* genotypes were analyzed for the presence of potential association with sperm parameters, no significant genotype-dependent differences were observed among infertile men (Table 4). However, among fertile men, *TGFB3* variant genotypes were associated with lower percentage of spermatozoa with progressive motility (reaching significance level for rs2268626), higher percentage of non-progressive motility (rs2268626, rs3917158, all the studied SNPs), as well as decreased total motility and higher percentage of immotile spermatozoa among variant carriers (rs2268626 and rs2284792). *TNF* rs1800629 variant was associated with lower percentage of spermatozoa with progressive motility. In infertile subjects the genetic associations and sperm parameters were mostly not observed.

## Discussion

The normal function of the blood–testis barrier protects developing germ cells against harmful agents and immunological influences. Protection is necessary because many agents or immune system cells can disturb the delicate process of meiotic cell division, thus leading to infertility. The barrier include structural component build of tight junctions as well as functional elements being mainly membrane transporters, e.g. glycoprotein P. TNF-α and TGF-β3 are two crucial cytokines, that are involved in the regulation of the blood-testis barrier, affecting both its structure as well as activity of functional components^6^. Therefore, the present study evaluated associations between polymorphisms of genes coding for TNF-α and TGF-β3, i.e. *TNF* and *TGFB3*, and male infertility.

Our study did not reveal significant association between *TNF* rs1800629 polymorphism and male infertility in a Polish population. This observation is contrary to the report of Tronchon *et al.* from French population, who revealed significantly increased frequency of the TNFα -308 A allele in infertile male patients with testicular failure or with altered sperm motility compared with patients with normal sperm parameters^17^. Indian population data published by Shukla *et al.* are in keeping with French observations. i.e. substitution level from G to A in the TNF-α gene was significantly higher in the infertile subjects as compared to fertile controls^18^. Likewise, Egyptian population report by Zalata *et al.* documented significant overrepresentation of *TNF* -308AA carriers among patients diagnosed with asthenozoospermia, asthenoteratozoospermia and oligoasthenoteratozoospermia^19^. The latter study revealed also that TNF-α -308AA genotype was significantly associated with decreased sperm count, sperm motility, normal sperm morphology in the above mentioned groups of infertile patients. Contrary, our data revealed that in the group of infertile patients any of the studied sperm parameters was not significantly associated the studied TNF-α gene polymorphism. In the control group a marked association was seen with lower percentage of spermatozoa with progressive motility.

To our knowledge, this is the first communication on the association between male infertility and *TGFB3* polymorphism. The results of the study demonstrated similar (borderline for rs2284792, p = 0.056) distribution of the studied *TGFB3* genotypes in infertile and fertile men. However, higher frequency of the minor alleles (in all the studied SNPs) in infertile men, being significant for *TGFB3* rs2284792. It was also found that heterozygous rs2284792 AG genotype was associated with significantly increased odds for infertility and similar results were observed for G allele carrier status. In the case of *TGFB3* rs3917158 a significant association with infertility in heterozygous subjects was found, with odd ratio of 1.37. Analysis in the subgroup of infertile men with semen abnormalities according to the WHO criteria (65.1% of infertile subjects) revealed a significant association of *TGFB3* rs2284792 variant with infertility; differences were significant for overall comparison, dominant model and allele frequency. Significant association for *TGFB3* rs3917158 SNP could also be observed for that subgroup analysis (p < 0.05 for dominant model and increased frequency of heterozygotes among infertile men with semen abnormalities, p = 0.056 for differences in minor allele frequency) (Supplementary Table 1). Those data may suggest that *TGFB3* polymorphism could be considered as a factor predisposing to male infertility. TGF-β3 is an important factor determining the blood-testis barrier integrity and its protective functions. Therefore, altered status for TGF-β3 generation produced by polymorphic variants in *TGFB3* gene may affect function and integrity of the blood-testis barrier, thus leading to infertility. However, confirmation from studies involving higher number of subjects as well as recruited in different populations is required.

The association of *TGFB3* polymorphism with male infertility can be also explained by other findings of the present study. It was found that the *TGFB3* polymorphism may affect sperm parameters. The study revealed that *TGFB3* polymorphism was associated with sperm motility parameters, i.e. *TGFB3* variant genotypes were associated with lower percentage of spermatozoa with progressive motility (reaching significance level for rs2268626), higher percentage of non-progressive motility (rs2268626, rs3917158, all the studied SNPs), as well as decreased total motility and higher percentage of immotile spermatozoa among fertile men variant allele carriers (rs2268626 and rs2284792). No associations were found in the whole infertile men group, possibly due to more pronounced dysfunction of spermatozoa. However, separate analysis of sperm parameters in normozoospermic infertile men and infertile men with semen abnormalities revealed no significant genotype-related differences, but lower percentage of motile spermatozoa was observed in *TGFB3* rs2268626 and rs2284792 minor homozygotes (Supplementary Table 2). That observation is in concordance with the results obtained for the control group of fertile men. There is no direct evidence from human studies on relationship between blood-testis barrier damage and spermatozoa-motility defects. However, it has been presented, that mice with disruption of blood-testis barrier integrity, induced by a high fat diet, were characterized by significantly decreased total and progressive motility of spermatozoa^20^. Similarly, administration of lipopolysaccharide with resultant disruption of blood-testis barrier, was associated with significant reduction in sperm count and spermatozoa motility in rats^21^. Other known disruptors of blood-testis barrier, i.e. TNF-α and IL-6, were also documented to significantly reduce spermatozoa progressive motility^22^,^23^. Those data may contribute to explanation of the observed associations between polymorphic variants in TGF-β3 and spermatozoa motility parameters. However, the results of the present study do not provide direct evidence that TGF-β3 may impact spermatozoa motility, only associations of *TGFB3* polymorphism and spermatozoa motility parameters were evidenced. As it was stated above, TGF-β3 may also *via* effects on the barrier function affect testicular microenvironment, and thus indirectly the motility.

In conclusion, the results of the present study may suggest an association between *TGFB3* polymorphism and male infertility. However, the observations should be verified in other studies, as the observed associations may result from a limited sample size (ethnic and environmental variables should also be addressed).

## Methods

### Subjects and study protocol

The study was carried out on 423 consecutive, otherwise healthy male patients, aged 32.9 ± 4.5 years (range: 19–52 years) without any chromosomal abnormalities, undergoing semen analysis due to infertility workup. The inclusion criteria were as following: age 18 to 56 years; no children from current or previous relations with at least a year history of at least a year of regular (2–3 weekly), unprotected sexual activity without conception; female partners aged up to 35 years with regular menstrual bleedings and/or progesterone levels in the luteal phase of the cycle >10 ng/ml, normal transvaginal ultrasound examination, negative testing for *Chlamydia trachomatis* infection, without history of pelvic inflammatory disease or abdominal operations. Male factor exclusion criteria included: clinical picture suggestive of obturatory azoospermia; history of testicular, epididymis or accessory gland infection; testicular torsio, maldescence or injury; varicocele; co-existing systemic disease; history of mumps.

The control group consisted of 423 healthy males, aged 34.3 ± 8.3 years, (range: 21–62) recruited among consecutive men accompanying their female partners at term labor at the Department of Reproductive Medicine and Gynecology, Siedlecka str., Police, Poland. Paternity was confirmed by women; however the possible paternal discrepancy was additionally checked based on blood group verification.

Both the men undergoing infertility workup as well as the fertile controls were of Polish origin, recruited within the same geographical region.

Semen samples were collected by masturbation after two to seven days of abstinence from sexual activity. Among infertile men, full seminograms, allowing for detailed data analysis were available for 344 patients. As for the fertile controls, that group was recruited among men accompanying their female partners at term labor, and only 124 of men from this group agreed to provide sperm sample for analysis (29.3%). However, that subgroup is representative for the whole study control group (no significant differences for age nor ethnicity) and provides necessary data on sperm parameters in healthy Polish males.

Sperm parameters were evaluated manually within one hour after the sample collection. Concentration, motility and morphology of spermatozoa were assessed according to the 2010 World Health Organization guidelines^24^. Sperm concentration was determined in an improved Neubauer haemocytometer (Heinz Hernez Medizinalbedarf GmbH, Hamburg, Germany), whereas sperm motility was analyzed under a phase-contrast microscope (Eclipse 200, Nikon, Japan), equipped with a heated stage. Sperm morphology was assessed, following the Papanicolaou staining of semen smears, under a bright light microscope (BX 41 Olympus Optical Co., Ltd., Tokyo, Japan) at 1000 × magnification. Study participants were categorized according to sperm concentration, motility and morphology as normozoospermic (with normal morphology) and subjects with abnormal standard sperm parameters (below the lower reference limits).

The study was approved by the Ethics Committee of Pomeranian Medical University (KB-0012/116/10) and conducted according to the Declaration of Helsinki. All participants provided written informed consent prior to participating in the study.

### Genotyping

All the subjects were genotyped for rs1800629 (−308G > A, MAF: 0.173) SNP in the promoter region of the *TNF*, previously associated with higher gene expression (A allele). Five tag SNPs with frequencies >0.1, mapping the *TGFB3* gene (rs2268626:T > C, MAF: 0.221; rs3917158:C > T, MAF: 0.199; rs2284792:A > G, MAF: 0.261; rs2268625:T > C, MAF: 0.190; rs3917187:C > T, MAF: 0.226) were selected with tag SNP picker using data for Caucasian population from the International HapMap Project (http://www.hapmap.org). MAFs are given for CEU population (Utah residents with Northern and Western European ancestry) from HapMap database. All the SNPs are located in gene introns. Genomic DNA was extracted from 200 mL of whole blood samples using Gene-MATRIX Quick Blood DNA Purification Kit (EURx, Poland). Commercially available, pre-validated allelic discrimination TaqMan real-time PCR assays (Life Technologies, USA) were used for genotyping. Fluorescence data was captured using ViiA 7 Real-Time PCR System (Life Technologies, USA) after 40 cycles of PCR.

### Statistical analysis

Genotype distribution was tested for Hardy-Weinberg equilibrium using Chi^2^ test. Continuous variables were compared between groups using Mann-Whitney U-test (comparison between two groups) or Kruskal-Wallis test (more than 2 groups). Associations between categorical variables were assessed by the Chi^2^ test, with Yate’s corrections for n < 5 (Statistica 10.0, Statsoft Software, Poland). The EH program (Jurg Ott, Rockefeller University, New York) was used to estimate haplotype frequencies. Linkage disequilibrium (LD) was measured: the D’ was calculated using 2LD software and squared correlation coefficient (r^2^) was evaluated. A p level of less than 0.05 was considered statistically significant.

## Additional Information

**How to cite this article**: Droździk, M. *et al.* TGFβ3 (*TGFB3*) polymorphism is associated with male infertility. *Sci. Rep.* **5**, 17151; doi: 10.1038/srep17151 (2015).

## Supplementary Material

Supplementary Information

## Figures and Tables

**Table 1:** Descriptive characteristics of standard sperm parameters of study subjects.

Sperm parameters	unit	Infertile men (n = 344)		Fertile men (n = 124)		p value
		Mean ± SD	% of normal values*	Mean ± SD	% of normal values	
Concentration	×10^6^/mL	39.9 ± 37.5	72.6	85.4 ± 72.8	94.4	<10^−5^
Total number of spermatozoa	×10^6^	167 ± 204	70.2	304 ± 299	97.2	<10^−5^
Morphologically normal spermatozoa	%	4.5 ± 4.7	43.5	10.2 ± 7.1	90.2	<10^−5^
Progressive motility	%	39.8 ± 21.8	62.2	47.5 ± 17.2	85.5	0.0007
Non-progressive motility	%	13.5 ± 10.4	–	18.8 ± 9.7	–	<10^−5^
Total motility	%	53.0 ± 20.0	77.6	66.4 ± 14.8	96.0	<10^−5^
Immotile spermatozoa	%	47.0 ± 20.0	–	33.6 ± 14.8	–	<10^−5^

SD–standard deviation; p value calculated by means of Mann-Whitney U-test; *according to the WHO guidelines.

**Table 2:** Frequency of the studied genotypes and alleles in infertile (n = 423) vs. fertile men (n = 423).

	Infertile patients		Fertile controls		p^1^	p^2^	p^3^	p^4^	p^5^
	n	(%)	n	(%)					
*TNF* rs1800629									
GG	312	(73.8)	301	(71.2)	0.507				
GA	105	(24.8)	118	(27.9)		0.568	0.330	0.397	0.525
AA	6	(1.4)	4	(0.9)					
MAF		(13.8)		(14.9)	0.389				
*TGFB3* rs2268626									
TT	275	(65.0)	298	(70.5)					
CT	134	(31.7)	111	(26.2)	0.214	0.836	0.079	0.090	1.000
CC	14	(3.3)	14	(3.3)					
MAF		(19.1)		(16.4)	0.143				
*TGFB3* rs3917158									
CC	287	(67.9)	311	(73.5)					
CT	127	(30.0)	100	(23.7)	0.101	0.643	0.041	0.070	0.507
TT	9	(2.1)	12	(2.9)					
MAF		(17.1)		(14.7)	0.162				
*TGFB3* rs2284792									
AA	240	(56.8)	274	(64.8)					
AG	163	(38.5)	133	(31.4)	0.056	0.303	0.021	0.017	0.496
GG	20	(4.7)	16	(3.8)					
MAF		(24.0)		(19.5)	0.025				
*TGFB3* rs2268625									
TT	288	(68.1)	311	(73.5)	0.176	0.966	0.064	0.082	0.825
TC	125	(29.6)	101	(23.9)					
CC	10	(2.3)	11	(2.6)	0.143				
MAF		(17.1)		(14.5)					
*TGFB3* rs3917187									
CC	257	(60.8)	282	(66.7)					
CT	149	(35.2)	125	(29.6)	0.192	0.669	0.071	0.074	0.859
TT	17	(4.0)	16	(3.8)					
MAF		(21.6)		(18.6)	0.115				

MAF – minor allele frequency; p values calculated by means of χ^2^ test, with Yate’s corrections for n < 5. p^1^ – overall comparison; p^2^ – major homozygotes vs. minor homozygotes; p^3^ – major homozygotes vs. heterozygotes; p^4^ – dominant model (minor homozygotes and heterozygotes vs. major homozygotes); p^5^ – recessive model (minor homozygotes vs. other genotypes);

**Table 3:** Frequency of *TGFB3* haplotypes in infertile (n = 423) and fertile men (n = 423).

*TGFB3* haplotype	Infertile patients	Fertile controls	p value
T_C_A_T_C	0.750	0.786	–
T_C_A_T_T	0.006	0.012	0.301
T_C_G_T_T	0.041	0.029	0.186
C_C_G_T_C	0.020	0.017	0.587
C_T_G_C_T	0.165	0.139	0.117
other	0.018	0.017	0.851

Haplotypes formed by 5 SNPs within *TGFB3* gene: rs2268626:T > C, rs3917158:C > T, rs2284792:A > G, rs2268625:T > C, rs3917187:C > T; p value calculated by means of Fisher exact test in relation to fertile controls, T_C_A_T_C as a referent haplotype. Haplotypes with estimated frequency > 1% in any study group are presented.

**Table 4:** Association between the studied *TGFB3/TNF* genotypes and sperm parameters in infertile (n = 344) and fertile (n = 124) men.

Sperm parameters	Unit	Group	*TNF* rs1800629 genotype			p value		
			GG	GA	AA	p^1^	p^2^	p^3^
Concentration	×10^6^/mL	infertile	39.7 ± 37.2	40.6 ± 38.9	33.7 ± 23.8	0.944	0.738	0.981
		fertile	82.1 ± 74.3	94.5 ± 67.5	ND	–	0.198	–
Morphologically normal spermatozoa	%	infertile	4.5 ± 4.8	4.5 ± 4.9	4.5 ± 4.8	0.981	0.851	0.929
		fertile	10.2 ± 7.3	10.7 ± 6.4	ND	–	0.513	–
Progressive motility	%	infertile	39.7 ± 21.9	40.1 ± 21.6	31.0 ± 16.7	0.611	0.959	0.338
		fertile	49.2 ± 16.8	40.1 ± 16.3	ND	–	0.035	–
Non-progressive motility	%	infertile	13.8 ± 10.2	13.0 ± 11.2	11.6 ± 6.4	0.585	0.303	0.776
		fertile	18.1 ± 9.2	22.1 ± 10.8	ND	–	0.084	–
Total motility	%	infertile	53.5 ± 19.9	52.2 ± 19.7	42.6 ± 12.0	0.357	0.516	0.166
		fertile	67.5 ± 14.6	62.1 ± 14.9	ND	–	0.221	–
Immotile spermatozoa	%	infertile	45.7 ± 19.9	45.4 ± 19.7	57.4 ± 12.0	0.335	0.796	0.140
		fertile	32.5 ± 14.6	37.8 ± 14.9	ND	–	0.230	–
**Sperm parameters**	**unit**		***TGFB3* rs2268626 genotype**			**p value**		
			**TT**	**CT**	**CC**	**p^1^**	**p^2^**	**p^3^**
Concentration	×10^6^/mL	infertile	40.8 ± 40.3	39.1 ± 32.0	27.4 ± 25.4	0.440	0.781	0.257
		fertile	87.9 ± 76.3	80.5 ± 69.7	65.4 ± 20.4	0.876	0.618	0.974
Morphologically normal spermatozoa	%	infertile	4.5 ± 4.9	4.6 ± 4.6	3.7 ± 4.9	0.487	0.654	0.238
		fertile	10.9 ± 7.3	9.5 ± 6.9	6.4 ± 2.1	0.326	0.226	0.239
Progressive motility	%	infertile	39.3 ± 21.8	41.5 ± 21.8	30.4 ± 19.0	0.208	0.673	0.120
		fertile	50.7 ± 17.8	40.1 ± 16.7	41.0 ± 10.6	0.024	0.006	0.469
Non-progressive motility	%	infertile	14.1 ± 10.9	13.0 ± 9.6	9.2 ± 5.5	0.326	0.272	0.207
		fertile	18.6 ± 10.3	18.3 ± 7.5	30.2 ± 8.2	0.034	0.289	0.010
Total motility	%	infertile	53.1 ± 19.5	54.4 ± 20.6	39.6 ± 18.8	0.414	0.924	0.016
		fertile	69.3 ± 14.6	58.9 ± 13.3	70.8 ± 9.6	0.002	0.002	0.465
Immotile spermatozoa	%	infertile	46.5 ± 19.4	43.7 ± 20.1	52.1 ± 21.6	0.156	0.471	0.124
		fertile	30.7 ± 14.6	41.1 ± 13.3	28.8 ± 9.8	0.002	0.002	0.427
**Sperm parameters**	**unit**		***TGFB3* rs3917158 genotype**			**p value**		
			**CC**	**CT**	**TT**	**p^1^**	**p^2^**	**p^3^**
Concentration	×10^6^/mL	infertile	41.4 ± 39.9	36.9 ± 32.1	32.1 ± 27.9	0.886	0.678	0.728
		fertile	80.1 ± 77.5	78.6 ± 63.9	65.5 ± 20.4	0.950	0.758	0.974
Morphologically normal spermatozoa	%	infertile	4.6 ± 4.9	4.3 ± 4.4	4.8 ± 5.8	0.642	0.357	0.716
		fertile	10.9 ± 7.6	9.1 ± 5.9	6.4 ± 2.1	0.326	0.214	0.238
Progressive motility	%	infertile	39.6 ± 21.6	40.2 ± 22.4	35.1 ± 19.7	0.785	0.961	0.507
		fertile	49.0 ± 18.6	42.8 ± 11.6	41.0 ± 10.6	0.308	0.131	0.469
Non-progressive motility	%	infertile	14.0 ± 10.7	13.0 ± 9.8	9.0 ± 5.3	0.326	0.253	0.231
		fertile	18.5 ± 10.1	18.4 ± 7.5	30.2 ± 8.2	0.034	0.245	0.010
Total motility	%	infertile	53.3 ± 19.4	53.2 ± 21.4	44.1 ± 16.9	0.331	0.827	0.139
		fertile	67.6 ± 15.9	61.8 ± 10.8	70.8 ± 9.6	0.085	0.083	0.466
Immotile spermatozoa	%	infertile	46.3 ± 19.3	43.9 ± 20.8	55.9 ± 16.9	0.196	0.645	0.110
		fertile	32.4 ± 15.9	38.2 ± 10.8	28.8 ± 9.8	0.085	0.085	0.427
**Sperm parameters**	**unit**		***TGFB3* rs2284792 genotype**			**p value**		
			**AA**	**AG**	**GG**	**p^1^**	**p^2^**	**p^3^**
Concentration	×10^6^/mL	infertile	40.6 ± 38.2	40.1 ± 37.5	27.8 ± 25.8	0.425	0.986	0.204
		fertile	93.0 ± 81.8	74.3 ± 58.5	60.7 ± 21.8	0.622	0.335	0.705
Morphologically normal spermatozoa	%	infertile	4.7 ± 5.1	4.2 ± 4.3	4.2 ± 5.0	0.467	0.251	0.475
		fertile	10.8 ± 6.8	10.0 ± 7.9	6.3 ± 1.9	0.270	0.194	0.203
Progressive motility	%	infertile	39.9 ± 22.3	40.3 ± 21.0	32.3 ± 21.8	0.311	0.871	0.131
		fertile	49.7 ± 16.5	44.5 ± 17.6	36.2 ± 15.1	0.114	0.060	0.171
Non-progressive motility	%	infertile	13.8 ± 10.9	13.5 ± 10.1	10.9 ± 6.5	0.797	0.748	0.506
		fertile	19.0 ± 9.7	17.4 ± 9.2	30.0 ± 7.4	0.012	0.989	0.004
Total motility	%	infertile	53.3 ± 20.1	53.8 ± 19.5	43.2 ± 20.4	0.094	0.760	0.031
		fertile	68.7 ± 14.0	62.3 ± 15.5	65.8 ± 14.9	0.086	0.034	0.985
Immotile spermatozoa	%	infertile	46.2 ± 20.1	44.7 ± 19.1	50.6 ± 21.5	0.304	0.868	0.149
		fertile	31.3 ± 13.9	37.7 ± 15.5	33.8 ± 15.1	0.084	0.035	0.938
**Sperm parameters**	**unit**		***TGFB3* rs2268625 genotype**			**p value**		
			**TT**	**TC**	**CC**	**p^1^**	**p^2^**	**p^3^**
Concentration	×10^6^/mL	infertile	41.3 ± 39.8	37.4 ± 32.2	29.1 ± 27.6	0.746	0.731	0.453
		fertile	86.3 ± 75.4	83.9 ± 71.8	65.5 ± 20.4	0.997	0.946	0.974
Morphologically normal spermatozoa	%	infertile	4.6 ± 4.9	4.4 ± 4.4	4.3 ± 5.6	0.656	0.456	0.488
		fertile	11.0 ± 7.6	9.1 ± 5.8	6.4 ± 2.1	0.349	0.247	0.238
Progressive motility	%	infertile	39.5 ± 21.7	40.5 ± 22.5	34.5 ± 18.5	0.698	0.896	0.435
		fertile	49.3 ± 18.7	42.4 ± 11.4	41.0 ± 10.6	0.183	0.067	0.469
Non-progressive motility	%	infertile	14.1 ± 10.7	12.8 ± 9.9	10.2 ± 6.2	0.358	0.178	0.426
		fertile	18.3 ± 10.2	18.9 ± 7.6	30.2 ± 8.2	0.027	0.134	0.010
Total motility	%	infertile	53.3 ± 19.4	53.2 ± 21.6	44.7 ± 16.0	0.322	0.836	0.135
		fertile	67.7 ± 16.1	61.8 ± 10.5	70.8 ± 9.6	0.070	0.065	0.465
Immotile spermatozoa	%	infertile	46.3 ± 19.3	43.8 ± 20.9	55.2 ± 15.9	0.180	0.632	0.105
		fertile	32.3 ± 16.1	38.2 ± 10.5	28.8 ± 9.8	0.066	0.068	0.427
**Sperm parameters**	**unit**		***TGFB3*** **rs3917187 genotype**			**p value**		
			**CC**	**CT**	**TT**	**p^1^**	**p^2^**	**p^3^**
Concentration	×10^6^/mL	infertile	40.0 ± 36.6	41.1 ± 40.1	28.0 ± 24.3	0.549	0.927	0.282
		fertile	88.1 ± 78.8	82.4 ± 66.1	60.7 ± 21.8	0.928	0.952	0.704
Morphologically normal spermatozoa	%	infertile	4.6 ± 4.7	4.4 ± 5.0	3.9 ± 4.7	0.522	0.293	0.503
		fertile	10.9 ± 7.2	9.9 ± 7.3	6.3 ± 1.9	0.317	0.255	0.203
Progressive motility	%	infertile	39.8 ± 22.0	40.0 ± 21.6	36.3 ± 20.7	0.749	0.876	0.449
		fertile	48.5 ± 17.7	46.5 ± 15.8	36.2 ± 15.1	0.274	0.232	0.171
Non-progressive motility	%	infertile	13.6 ± 10.6	13.6 ± 10.6	12.7 ± 5.8	0.853	0.802	0.676
		fertile	18.3 ± 9.3	18.7 ± 9.9	30.0 ± 7.4	0.017	0.330	0.004
Total motility	%	infertile	53.0 ± 20.2	53.5 ± 19.7	49.0 ± 19.0	0.464	0.908	0.222
		fertile	66.8 ± 15.7	65.5 ± 13.2	65.8 ± 14.9	0.727	0.450	0.986
Immotile spermatozoa	%	infertile	44.4 ± 20.1	44.0 ± 19.1	51.0 ± 19.0	0.276	0.676	0.167
		fertile	33.2 ± 15.7	34.5 ± 13.2	33.8 ± 15.1	0.721	0.457	0.939

Mean values and standard deviation are presented; ND - genotypes were not detected in subjects with sperm parameters available; p^1^ – overall comparison – Kruskal-Wallis test; p^2^ – dominant model (minor homozygotes and heterozygotes vs. major homozygotes) – U-test; p^3^ –recessive model (minor homozygotes vs. other genotypes) -U-test.
