# Supplementary material for: TGFβ3 (TGFB3) polymorphism is associated with male infertility
Source: Sci Rep. 2015 Nov 27;5:17151. doi: 10.1038/srep17151 (PMC4661604; doi:10.1038/srep17151)
Supplement: Supplementary Information [file srep17151-s1.doc]

**TGFβ3 (*TGFB3*) polymorphism is associated with male infertility**

Marek Droździk, Maciej Kaczmarek, Damian Malinowski, Urszula Broś, Anna Kazienko, Rafał Kurzawa, Mateusz Kurzawski

|  | Infertile patients | | Fertile controls | | p^1^ | p^2^ | p^3^ | p^4^ | p^5^ |
| --- | --- | --- | --- | --- | --- | --- | --- | --- | --- |
|  | n | (%) | n | (%) |  |  |  |  |  |
| *TNF* rs1800629 |  | | | | | | | | |
| GG | 163 | (72.8) | 301 | (71.2) |  |  |  |  |  |
| GA | 56 | (25.0) | 118 | (27.9) | 0.324 | 0.359 | 0.485 | 0.665 | 0.328 |
| AA | 5 | (2.2) | 4 | (0.9) |  |  |  |  |  |
| MAF |  | (14.7) |  | (14.9) | 0.938 |  |  |  |  |
| *TGFB3* rs2268626 |  | | | | | | | | |
| TT | 146 | (65.2) | 298 | (70.5) |  |  |  |  |  |
| CT | 69 | (30.8) | 111 | (26.2) | 0.387 | 0.535 | 0.194 | 0.169 | 0.643 |
| CC | 9 | (4.0) | 14 | (3.3) |  |  |  |  |  |
| MAF |  | (19.4) |  | (16.4) | 0.178 |  |  |  |  |
| *TGFB3* rs3917158 |  | | | | | | | | |
| CC | 151 | (67.4) | 311 | (73.5) |  |  |  |  |  |
| CT | 68 | (30.4) | 100 | (23.7) | 0.172 | 0.777 | 0.069 | 0.101 | 0.647 |
| TT | 5 | (2.2) | 12 | (2.9) |  |  |  |  |  |
| MAF |  | (17.4) |  | (14.7) | 0.194 |  |  |  |  |
| *TGFB3* rs2284792 |  | | | | | | | | |
| AA | 121 | (54.0) | 274 | (64.8) |  |  |  |  |  |
| AG | 91 | (40.6) | 133 | (31.4) | 0.027 | 0.178 | 0.012 | 0.008 | 0.349 |
| GG | 12 | (5.4) | 16 | (3.8) |  |  |  |  |  |
| MAF |  | (25.7) |  | (19.5) | 0.010 |  |  |  |  |
| *TGFB3* rs2268625 |  | | | | | | | | |
| TT | 152 | (67.9) | 311 | (73.5) |  |  |  |  |  |
| TC | 66 | (29.5) | 101 | (23.9) | 0.296 | 0.832 | 0.119 | 0.117 | 0.952 |
| CC | 6 | (2.6) | 11 | (2.6) |  |  |  |  |  |
| MAF |  | (17.4) |  | (14.5) | 0.175 |  |  |  |  |
| *TGFB3* rs3917187 |  | | | | | | | | |
| CC | 130 | (58.5) | 282 | (66.7) |  |  |  |  |  |
| CT | 84 | (37.0) | 125 | (29.6) | 0.093 | 0.463 | 0.032 | 0.030 | 0.674 |
| TT | 10 | (4.5) | 16 | (3.8) |  |  |  |  |  |
| MAF |  | (23.2) |  | (18.6) | 0.058 |  |  |  |  |

**Supplementary Table 1. Frequency of the studied genotypes and alleles in infertile men with confirmed semen abnormalities (n=224) vs. fertile men (n=423).** MAF – minor allele frequency; p values calculated by means of χ^2^ test , with Yate’s corrections for n<5. p^1^ – overall comparison; p^2^ – major homozygotes vs. minor homozygotes; p^3^ – major homozygotes vs. heterozygotes; p^4^ – dominant model (minor homozygotes and heterozygotes vs. major homozygotes); p^5^ – recessive model (minor homozygotes vs. other genotypes);

| Sperm parameters | Unit | Semen analysis | *TNF* rs1800629 genotype | | | p value | | |
| --- | --- | --- | --- | --- | --- | --- | --- | --- |
|  |  |  | GG | GA | AA | p^1^ | p^2^ | p^3^ |
| Concentration | x10^6^ /mL | normal | 62.9±37.8 | 58.1±29.3 | - | 0.740 | 0.742 | - |
|  |  | abnormal | 26.9±30.3 | 31.5±40.6 | 33.8±23.8 | 0.431 | 0.312 | 0.301 |
| Morphologically normal spermatozoa | % | normal | 9.0±4.9 | 7.8±3.2 | - | 0.468 | 0.475 | - |
|  |  | abnormal | 2.4±2.8 | 3.1±4.9 | 3.8±2.8 | 0.300 | 0.334 | 0.171 |
| Progressive motility | % | normal | 59.8±13.6 | 59.0±13.7 | - | 0.819 | 0.821 | - |
|  |  | abnormal | 28.6±17.4 | 30.4±18.6 | 31.0±16.8 | 0.711 | 0.410 | 0.831 |
| Non-progressive motility | % | normal | 10.3±7.2 | 11.4±15.1 | - | 0.281 | 0.284 | - |
|  |  | abnornaml | 15.8±11.1 | 13.9±8.8 | 11.6±6.4 | 0.548 | 0.377 | 0.400 |
| Total motility | % | normal | 70.1±11.4 | 67.8±11.4 | - | 0.394 | 0.396 | - |
|  |  | abnormal | 44.4±17.8 | 44.4±19.1 | 42.6±12.0 | 0.958 | 0.962 | 0.793 |
| Immotile spermatozoa | % | normal | 29.9±11.4 | 32.3±11.4 | - | 0.383 | 0.396 | - |
|  |  | abnormal | 54.4±18.2 | 52.1±19.9 | 57.4±12.0 | 0.754 | 0.611 | 0.685 |
|  | | | *TGFB3* rs2268626 genotype | | | p value | | |
| Sperm parameters | unit |  | TT | CT | CC | p^1^ | p^2^ | p^3^ |
| Concentration | x10^6^ /mL | normal | 65.2±37.2 | 54.7±33.9 | 63.7±13.9 | 0.207 | 0.177 | 0.415 |
|  |  | abnormal | 27.9±36.1 | 30.6±27.6 | 15.3±13.3 | 0.227 | 0.329 | 0.277 |
| Morphologically normal spermatozoa | % | normal | 8.3±4.7 | 9.5±4.2 | 10.7±5.8 | 0.207 | 0.084 | 0.477 |
|  |  | abnormal | 2.8±3.9 | 2.4±2.7 | 1.3±0.9 | 0.373 | 0.447 | 0.191 |
| Progressive motility | % | normal | 60.1±12.6 | 58.9±15.5 | 55.0±15.1 | 0.776 | 0.593 | 0.572 |
|  |  | abnormal | 28.2±17.3 | 31.9±18.7 | 22.2±11.6 | 0.177 | 0.266 | 0.264 |
| Non-progressive motility | % | normal | 10.9±10.5 | 10.5±8.0 | 4.7±5.0 | 0.359 | 0.797 | 0.156 |
|  |  | abnornaml | 15.9±10.8 | 14.4±10.2 | 10.7±5.1 | 0.328 | 0.209 | 0.264 |
| Total motility | % | normal | 70.0±10.1 | 69.4±13.5 | 59.7±11.5 | 0.349 | 0.663 | 0.151 |
|  |  | abnormal | 44.1±17.3 | 46.2±19.3 | 32.9±15.8 | 0.062 | 0.556 | 0.043 |
| Immotile spermatozoa | % | normal | 29.9±10.1 | 30.6±13.5 | 40.311.5 | 0.349 | 0.652 | 0.151 |
|  |  | abnormal | 55.2±17.5 | 50.9±19.7 | 56.0±23.2 | 0.131 | 0.174 | 0.297 |
|  | | | *TGFB3* rs3917158 genotype | | | p value | | |
| Sperm parameters | unit |  | CC | CT | TT | p^1^ | p^2^ | p^3^ |
| Concentration | x10^6^ /mL | normal | 65.2±36.8 | 53.6±34.3 | 63.7±13.9 | 0.149 | 0.129 | 0.415 |
|  |  | abnormal | 28.6±35.7 | 28.5±27.6 | 13.1±8.1 | 0.643 | 0.793 | 0.412 |
| Morphologically normal spermatozoa | % | normal | 8.4±4.7 | 9.3±4.1 | 10.7±5.8 | 0.371 | 0.179 | 0.477 |
|  |  | abnormal | 2.8±3.9 | 2.3±2.7 | 1.2±0.8 | 0.320 | 0.274 | 0.229 |
| Progressive motility | % | normal | 59.8±12.3 | 59.6±16.3 | 55.0±15.1 | 0.848 | 0.917 | 0.572 |
|  |  | abnormal | 28.7±17.5 | 30.5±18.5 | 23.2±9.7 | 0.532 | 0.514 | 0.456 |
| Non-progressive motility | % | normal | 11.0±10.4 | 10.1±8.1 | 4.7±5.0 | 0.324 | 0.438 | 0.156 |
|  |  | abnornaml | 15.7±10.7 | 14.4±10.4 | 11.6±3.8 | 0.536 | 0.318 | 0.488 |
| Total motility | % | normal | 69.8±9.9 | 69.7±14.1 | 59.7±11.5 | 0.346 | 0.873 | 0.151 |
|  |  | abnormal | 44.4±17.4 | 44.9±19.6 | 34.8±12.0 | 0.255 | 0.743 | 0.132 |
| Immotile spermatozoa | % | normal | 30.1±10.0 | 30.3±14.1 | 40.3±11.5 | 0.347 | 0.861 | 0.151 |
|  |  | abnormal | 54.9±17.6 | 50.7±20.3 | 65.2±12.0 | 0.076 | 0.247 | 0.099 |

**Supplementary table 2. Association between the studied *TGFB3/TNF* genotypes and sperm parameters in two subgroups of infertile men: normozoospermic (n=120) and with confirmed semen abnormalities (n=224).**Mean values and standard deviation are presented; ND- genotypes were not detected in subjects with sperm parameters available;p^1^ – overall comparison – Kruskal-Wallis test; p^2^ – dominant model (minor homozygotes and heterozygotes vs. major homozygotes) – U-test; p^3^ –recessive model (minor homozygotes vs. other genotypes) -U-test.

|  | | | *TGFB3* rs2284792 genotype | | | | p value | | | |
| --- | --- | --- | --- | --- | --- | --- | --- | --- | --- | --- |
| Sperm parameters | unit |  | AA | AG | | GG | p^1^ | p^2^ | | p^3^ |
| Concentration | x10^6^ /mL | normal | 66.3±37.7 | 54.0±33.4 | | 66.5±12.7 | 0.100 | 0.136 | | 0.249 |
|  |  | abnormal | 25.7±30.1 | 33.4±37.7 | | 14.9±11.8 | 0.128 | 0.220 | | 0.216 |
| Morphologically normal spermatozoa | % | normal | 8.5±4.8 | 8.8±4.1 | | 11.5±5.0 | 0.371 | 0.402 | | 0.199 |
|  |  | abnormal | 2.8±4.1 | 2.5±2.8 | | 1.8±1.4 | 0.704 | 0.554 | | 0.483 |
| Progressive motility | % | normal | 60.3±13.0 | 58.3±14.4 | | 61.3±17.6 | 0.700 | 0.479 | | 0.785 |
|  |  | abnormal | 27.9±17.7 | 31.6±18.0 | | 22.7±12.3 | 0.116 | 0.214 | | 0.202 |
| Non-progressive motility | % | normal | 11.1±10.9 | 10.3±7.6 | | 5.0±4.2 | 0.350 | 0.740 | | 0.151 |
|  |  | abnornaml | 15.1±10.6 | 15.1±10.8 | | 12.8±6.0 | 0.828 | 0.584 | | 0.685 |
| Total motility | % | normal | 70.3±10.3 | 68.6±12.6 | | 66.3±16.2 | 0.684 | 0.420 | | 0.623 |
|  |  | abnormal | 43.3±17.9 | 46.7±18.2 | | 35.5±15.4 | 0.046 | 0.286 | | 0.049 |
| Immotile spermatozoa | % | normal | 29.7±10.4 | 31.4±12.6 | | 33.8±16.2 | 0.676 | 0.412 | | 0.623 |
|  |  | abnormal | 55.7±18.2 | 51.1±18.4 | | 56.2±20.6 | 0.081 | 0.107 | | 0.279 |
|  | | | *TGFB3* rs2268625 genotype | | | | p value | | | |
| Sperm parameters | unit |  | TT | TC | CC | | p^1^ | p^2^ | p^3^ | |
| Concentration | x10^6^ /mL | normal | 65.2±36.8 | 53.6±34.3 | 63.7±13.9 | | 0.149 | 0.129 | 0.415 | |
|  |  | abnormal | 28.5±35.6 | 29.1±27.8 | 11.8±8.0 | | 0.431 | 0.789 | 0.244 | |
| Morphologically normal spermatozoa | % | normal | 8.4±4.7 | 9.3±4.1 | 10.7±5.8 | | 0.371 | 0.179 | 0.477 | |
|  |  | abnormal | 2.8±3.9 | 2.4±2.7 | 1.2±0.8 | | 0.302 | 0.367 | 0.163 | |
| Progressive motility | % | normal | 59.8±12.3 | 59.6±16.3 | 55.0±15.1 | | 0.848 | 0.917 | 0.572 | |
|  |  | abnormal | 28.6±17.5 | 30.6±18.5 | 24.3±9.1 | | 0.567 | 0.474 | 0.556 | |
| Non-progressive motility | % | normal | 11.0±10.4 | 10.1±8.1 | 4.7±5.0 | | 0.324 | 0.438 | 0.156 | |
|  |  | abnornaml | 15.8±10.7 | 14.1±10.4 | 13.0±4.9 | | 0.465 | 0.217 | 0.755 | |
| Total motility | % | normal | 69.8±9.9 | 69.7±14.1 | 59.7±11.5 | | 0.346 | 0.873 | 0.151 | |
|  |  | abnormal | 44.4±17.4 | 44.7±19.8 | 37.3±12.4 | | 0.391 | 0.781 | 0.218 | |
| Immotile spermatozoa | % | normal | 30.1±10.0 | 30.3±14.1 | 40.3±11.5 | | 0.347 | 0.861 | 0.151 | |
|  |  | abnormal | 54.9±17.6 | 50.7±20.5 | 62.7±12.4 | | 0.123 | 0.263 | 0.165 | |
|  | | | *TGFB3* rs3917187 genotype | | | | p value | | | |
| Sperm parameters | unit |  | CC | CT | TT | | p^1^ | p^2^ | p^3^ | |
| Concentration | x10^6^ /mL | normal | 65.0±36.2 | 56.2±36.7 | 56.2±20.2 | | 0.368 | 0.175 | 1.000 | |
|  |  | abnormal | 25.6±28.6 | 34.0±39.9 | 13.9±8.7 | | 0.247 | 0.353 | 0.271 | |
| Morphologically normal spermatozoa | % | normal | 8.2±4.0 | 9.5±5.5 | 10.7±5.8 | | 0.490 | 0.279 | 0.477 | |
|  |  | abnormal | 2.8±3.9 | 2.5±3.0 | 1.9±1.4 | | 0.793 | 0.523 | 0.730 | |
| Progressive motility | % | normal | 59.6±12.8 | 59.7±14.8 | 58.4±17.5 | | 0.979 | 0.989 | 0.854 | |
|  |  | abnormal | 28.4±17.9 | 30.8±17.9 | 25.2±11.0 | | 0.442 | 0.380 | 0.505 | |
| Non-progressive motility | % | normal | 11.3±10.7 | 9.3±7.8 | 9.6±6.8 | | 0.439 | 0.251 | 0.833 | |
|  |  | abnornaml | 15.0±10.3 | 15.6±11.3 | 14.3±4.9 | | 0.982 | 0.897 | 0.869 | |
| Total motility | % | normal | 69.9±10.3 | 69.0±12.8 | 68.0±17.1 | | 0.927 | 0.749 | 0.770 | |
|  |  | abnormal | 43.4±18.2 | 46.4±18.3 | 39.5±11.4 | | 0.152 | 0.248 | 0.228 | |
| Immotile spermatozoa | % | normal | 30.0±10.3 | 31.0±12.8 | 32.0±17.1 | | 0.932 | 0.738 | 0.770 | |
|  |  | abnormal | 55.8±18.5 | 50.0±18.6 | 60.5±11.4 | | 0.052 | 0.075 | 0.159 | |

**Supplementary table 2 - continuation. Association between the studied *TGFB3/TNF* genotypes and sperm parameters in two subgroups of infertile men: normozoospermic (n=120) and with confirmed semen abnormalities (n=224).**Mean values and standard deviation are presented; ND- genotypes were not detected in subjects with sperm parameters available;p^1^ – overall comparison – Kruskal-Wallis test; p^2^ – dominant model (minor homozygotes and heterozygotes vs. major homozygotes) – U-test; p^3^ –recessive model (minor homozygotes vs. other genotypes) -U-test.
